# Exploring the combination and modular characteristics of herbs for alopecia treatment in traditional Chinese medicine: an association rule mining and network analysis study

**DOI:** 10.1186/s12906-018-2269-7

**Published:** 2018-07-04

**Authors:** Jungtae Leem, Wonmo Jung, Yohwan Kim, Bonghyun Kim, Kyuseok Kim

**Affiliations:** 1 Dongshin Korean Medicine Hospital, 351, Omok-ro, Yangcheon-gu, Seoul, 07999 Republic of Korea; 2 Chung-Yeon Medical Institute, 64, Sangmujungang-ro, Seo-gu, Gwangju, 61949 Republic of Korea; 3 Acupuncture and Meridian Science Research Center, College of Korean Medicine, Kyung Hee University, 26 Kyungheedae-ro, Dongdaemun-gu, Seoul, 02447 Republic of Korea; 4 Department of Science in Korean Medicine, Graduate School, Kyung Hee University, 26 Kyungheedae-ro, Dongdaemun-gu, Seoul, 02447 Republic of Korea; 5 College of Korean Medicine, Kyung Hee University, 26 Kyungheedae-ro, Dongdaemun-gu, Seoul, 02447 Republic of Korea; 6 Department of Clinical Korean medicine, Graduate School, Kyung Hee University, 26 Kyungheedae-ro, Dongdaemun-gu, Seoul, 02447 Republic of Korea; 7 Department of Ophthalmology, Otolaryngology & Dermatology, College of Korean Medicine, Kyung Hee University, 26 Kyungheedae-ro, Dongdaemun-gu, Seoul, 02447 Republic of Korea; 8 Department of Ophthalmology, Otolaryngology & Dermatology, Kyung Hee University Korean Medicine Hospital, 23 Kyungheedae-ro, Dongdaemun-gu, Seoul, 02447 Republic of Korea

**Keywords:** Medicinal herb, Alopecia, Association rule mining, Network analysis, Bioinformatics

## Abstract

**Background:**

Although alopecia affects the quality of life, its pathogenesis is unknown, because cellular interactions in the hair follicle are complex. Several authors have suggested using herbal medicine to treat alopecia, and bioinformatics and network pharmacology may constitute a new research strategy in this regard because herbal medicines contain various chemical components. This study used association rule mining (ARM) and network analysis to analyze the combinations of medicinal herbs used to treat alopecia.

**Methods:**

We searched Chinese, Korean, and English databases for literature about alopecia treatment, extracting the names of each herbal prescription and herb. The meridian tropism and classification category of each herb were also investigated. Using ARM, we identified frequently combined two-herb and three-herb sets. Using network analysis, we divided the herbs into several modules according to prescription pattern.

**Results:**

Fifty-six articles and 489 herbal medicines were included—312 internal and 177 external medicines. Among the 312 medicinal herbs used in internal medicine group, the most frequently combined two-herb set was *Polygonum multiflorum* Thunb. (何首烏) and *Angelica sinensis* (Oliv.) Dlels (當歸). The most frequently used three-herb combination was *Polygonum multiflorum* Thunb., *Angelica sinensis* (Oliv.) Dlels, and *Ligusticum chuanxiong* Hort. (川芎). In network analysis, three modules were identified. The herbs of Module 1 were related to the liver and kidney meridians, and those of Module 3 were related to the Stomach meridian.

**Conclusions:**

We identified the frequency, characteristics, and functional modules of herb combinations frequently used in alopecia treatment. We confirmed the value of classical medicinal herb theory. This finding will prompt further bioinformatics and network pharmacology research on alopecia.

**Electronic supplementary material:**

The online version of this article (10.1186/s12906-018-2269-7) contains supplementary material, which is available to authorized users.

## Background

Hair loss affects up to 50% of both men and women throughout their lives, causing anxiety and disability that can have a significant effect on the patient’s quality of life. [1, 2] The condition has been linked to an autoimmune disorder of the hair follicle, genetic background, hormones, medication, and psychological stress, which can alter the hair follicle cycle [3, 4] Many studies have attempted to elucidate the pathogenesis of hair loss. However, the complex molecular interactions between the cells of the hair follicle have not been fully understood, and the exact cause of alopecia is still unknown.

Finasteride and minoxidil have been approved by the Food and Drug Administration of the United States (FDA, USA) to promote hair growth. However, the effectiveness of these drugs varies greatly among individuals, and they have unwanted side effects. Relatedly, many alopecia patients are concerned about the side effects associated with conventional therapies, and complementary and alternative medicine (CAM) has thus been suggested as a new treatment for alopecia. [5] In particular, traditional Chinese medicine (TCM) is an important part of healthcare in East Asia, and it is commonly used to treat alopecia patients. [5, 6]

Herbal medicine prescriptions consist of various herbal preparations. Thus, using the scientific method, researchers must investigate frequently used herbal combinations and categorize them. However, in the TCM literature, few narrative reviews have focused on herbs for alopecia treatment, [6, 7] and the Chinese, Korean, and European research databases contain no studies that have classified herbs for alopecia treatment using statistical methods such as data mining.

Previous studies based on TCM pattern identification have shown that deficiency of liver and kidney (肝腎不足), deficiency of *qi* and blood (氣血兩虛), *qi* stagnation and blood stasis (氣滯血瘀), and blood-heat (血熱) are the main patterns linked to alopecia. [6, 7] However, because a diverse range of herbal ingredients are used in TCM and because interactions between herbal medicine and the human body are complex, the mechanism underlying these TCM patterns is still unknown. [8] Recently, statistical methods such as data mining have been applied to TCM research. However, to the best of our knowledge, no studies have used network analysis methods to assess herbal medicine used in hair loss treatment.

Therefore, this study aimed to identify—using association rule mining (ARM)—which herbal combinations are used frequently in hair loss treatment and to analyze the modular characteristics of these treatments using network analysis.

## Methods

### Criteria for study inclusion

We included all kinds of studies regardless of the study design, and we did not restrict the type of alopecia or herbal medicine used. Thus, all kinds of herbal medicines were considered—extracts, decoctions, pills, and even external application. There were no restrictions on sex, age, disease duration, or disease severity. The outcome of clinical studies was not considered.

### Search methods

We conducted an electronic search of the Chinese, English, and Korean databases from their inception to March 2017. We searched one Chinese database: the China National Knowledge Infrastructure (CNKI) database, three English databases: Embase, Medline (via PubMed), and the Central Register of Controlled Trials (CENTRAL), and one Korean database: the Oriental Medicine Advanced Searching Integrated System (OASIS), which specializes in traditional Korean Medicine research articles. [9] The following search terms for alopecia were included: “Alopecia”, “Alopecia areata”, “Diffuse alopecia”, “Androgenic alopecia,” and “Female pattern hair loss.” An additional file describes details of the search terms and search strategies used in each database to identify alopecia (see Additional file 1).

### Data extraction

We extracted the name of each herbal formula, the medicinal herbs that comprised it, its origin (name of article or ancient literature), author, publication year, and internal/external application. The names of the herbs followed the *Chinese Pharmacopoeia 2015 edition*, [8, 10] which can be found on the OASIS and KIOM Herbarium website (http://boncho.kiom.re.kr/herbarium/codex.php). [11] The categorization of each herb followed *Phytology* [12] and *Chinese Pharmacy*. [13] The names of the 20 classification categories of medicinal herbs are as follows: (1) Exterior-releasing medicinal (解表藥), (2) Heat-clearing medicinal (淸熱藥), (3) Purgative medicinal (瀉下藥), (4) Wind-dampness dispelling medicinal (祛風濕藥), (5) dampness-resolving medicinal (化濕藥), (6) Dampness-draining diuretic medicinal (利水滲濕藥), (7) Interior-warming medicinal (溫裏藥), (8) *Qi*-regulating medicinal (理氣藥), (9) Digestant medicinal (消食藥), (10) worm-expelling medicinal (驅蟲藥), (11) hemostatic (medicinal) (止血藥), (12) Blood-activating and stasis-dispelling medicinal (活血祛瘀藥), (13) Cough-suppressing and panting-calming medicinal (止咳平喘藥), (14) Tranquillizing medicinal (安神藥), (15) Liver-pacifying medicinal (平肝藥), (16) Orifice-opening medicinal (開竅藥), (17) Tonifying and replenishing medicinal (補益藥), (18) Astringent medicinal (收澁藥), (19) Emetic medicinal (湧吐藥), and (20) External application medicinal (外用藥).

### Data analysis

First, we compared the meridian tropism and classification category of each medicinal herb between the internal and external applications. The criteria for meridian tropism followed the classification of the *Phytology* [12] and *Chinese Pharmacy*. [13] Next, we conducted a data mining analysis using ARM and network analysis. Because the mechanism of action differs depending on the route of administration, we only carried out this analysis on the internal application formulas only, not the external application formulas. Using ARM, we identified the most frequently used two-herb combination and three-herb combination. Using network analysis, we categorized the herbs used in alopecia treatment into several modules.

### Association rule mining

Using the list of prescriptions used to treat hair loss, we searched for combinations of herbs repeatedly used over several prescriptions. For this purpose, we applied ARM, which uncovers interesting relationships in large datasets, to our data. [14] Because ARM is generally used in business to analyze customers’ purchase data, the terms “item” and “transaction” are widely used. In our analysis, the herbs were defined as items, and the prescriptions were defined as transactions recording co-occurrences of items. We let H = {h1, h2, …hd} be the set of all herbs in the bald prescription data, and P = {p1, p2, …, pn} be the set of all prescriptions. In ARM, a collection of zero or more items is termed an itemset. An association rule is an expression of the form X → Y, where X and Y are disjoint itemsets. The expression represents the relationship between the occurrences of itemset X and itemset Y. The strength of the association rule can be measured in terms of its support, confidence, and lift. Support determines how often a rule is applicable to a given data set, while confidence determines how frequently items in Y appear in transactions that contain X. Support indicates how frequently the rule can be applied to a given set of data, and confidence indicates how often Y appears in transactions containing X. Lift is the ratio of observed support to expected support when X and Y are independent. Support is a measure of whether an association between X and Y happens by chance, and confidence represents the reliability of the association. Lift values larger than 1 indicate that the occurrences the two itemsets are dependent on each other. These measures suggest a strong co-occurrence relationship between itemsets X and Y. In the present study, ARM for combinations of two herbs and three herbs was applied using the a priori package of R (R Core Team (2013). R: A language and environment for statistical computing. R Foundation for Statistical Computing, Vienna, Austria), and minimum thresholds on support and confidence were set at 0 and 1, respectively.

### Network analysis

ARM cannot inspect the overall pattern of how herbs are used together, because it assesses the association between limited numbers of itemsets. Therefore, we constructed a network that connected the herbs used together in alopecia prescriptions. We also examined the modularity analysis to identify patterns and group herbs into specific modules. The network between the herbs was configured using Python’s networkx® package (https://networkx.github.io/). [15] The nodes of the network were defined as all the herbs that appeared in alopecia prescriptions. The herbs that appeared together in at least one prescription were defined as having a linkage between each other, and the network was constructed as a graph that weighted connections based on the number of co-occurrences in different prescriptions. The dose of the herb in each prescription was not considered in determining weight or linkage. Modularity analysis and network visualization were performed using Gephi. [16] Modularity analysis was performed using the Louvaine method, with a resolution value of 1.0 [17]. Visualization was performed using a circular layout in which modules were classified into categories.

To observe differences among the modules identified modularity analysis, the meridian tropism of the herbs composing each module was examined. Meridian tropism is the notion that a herb predominantly exerts a therapeutic effect on a specific organ or meridian in the human body [18]. The meridian tropism (引經) of each herb is recorded in the classic book, and it reveals the characteristics of each herb from the perspective of Korean medicine. Based on the meridian tropism of each herb listed in herbal textbooks and the Korean Intellectual Property Office database, we investigated the ratios of therapeutic preferences for each meridian of the herbs in each module. The permutation test was then applied to find statistically significant meridian preferences. Briefly, a list of module labels of herbs was randomly permutated, the meridian preference ratio per module was calculated, and the process was repeated 10,000 times to obtain a null distribution of meridian preference ratios. A *p*-value was then calculated based on the location of the observations within the simulated null distribution. We tested 12 meridian preferences separately for each module, with a correction for multiple testing using the false discovery rate.

## Results

### Study selection

**Fig. 1:**
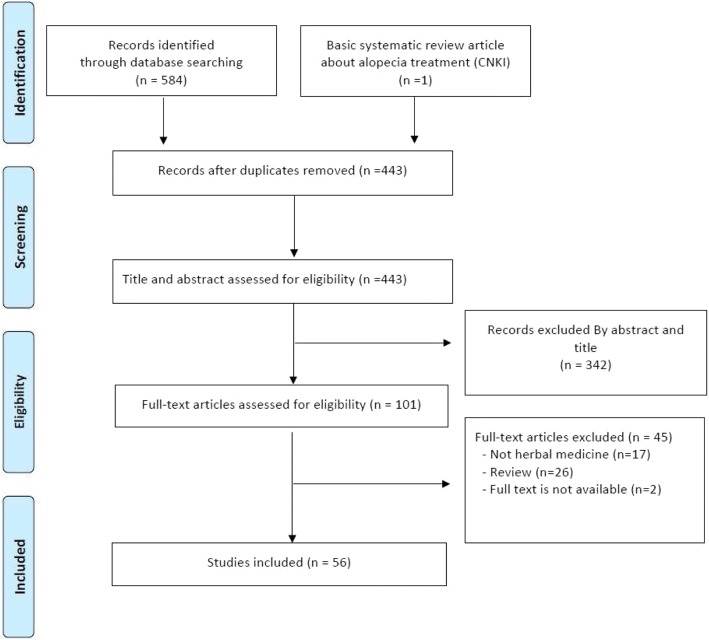
PRISMA flow diagram

A total of 585 articles were screened: 286 in the English databases, 73 in CNKI, and 226 in OASIS. Ultimately, 56 articles were included after screening of the full text: 12 from the English databases, [19–30], 13 from CNKI, [6, 31–42] and 31 from OASIS. [43–73] Details of the screening process are shown in the PRISMA flow chart (Fig. 1).

### Herbal medicine and medicinal herbs

From the 56 articles, 489 herbal medicines (312 internal and 177 external) and 374 medicinal herbs were identified. Among the 312 internal medicines, 258 medicinal herbs were identified. Among the 177 external medicines, 257 medicinal herbs were identified. Many medicinal herbs were used in both internal medicine and external applications. Table 1 lists the frequently described medicinal herbs.

**Table 1:** Frequency, module, and meridian tropism of the top 10 internal and external medicinal herbs

Internal/External	Medicinal Herb	Category	Frequency	Module	LR (肝)	HT (心)	PC (心包)	SP (脾)	LU (肺)	KI (腎)	GB (膽)	SI (小腸)	TE (三焦)	ST (胃)	LI (大腸)	BL (膀胱)
I&E	*Angelica sinensis* (Oliv.) Dlels (當歸)	17	171 (I) 27 (E)	1	Y	Y		Y								
I&E	*Ligusticum chuanxiong* Hort. (川芎)	12	132 (I) 32 (E)	1	Y		Y				Y					
I	*Polygonum multiflorum* Thunb. (何首烏)	17	175	1	Y					Y						
I	*Rehmannia glutinosa* Libosch. (Prepared) (熟地黃)	17	137	1	Y					Y						
I	*Poria cocos* (Schw.) Wolf (茯苓)	6	103	3		Y		Y		Y						
I	*Ligustrum lucidum* Ait. (女貞子)	17	96	1	Y					Y						
I	*Rehmannia glutinosa* Libosch. (生地黄)	2	95	1	Y	Y				Y						
I	*Glycyrrhiza uralensis* Fisch. (甘草)	8	90	3												
I	*Eclipta prostrata* L. (旱蓮草)	17	89	1	Y					Y						
I	*Lycium barbarum* L. (枸杞子)	17	88	1	Y				Y	Y						
E	*Platycladus orientalis* (L.) Franco (側柏葉)	11	51	1	Y				Y						Y	
E	*Angelica dahurica* (Fisch. ex Hoffm.) Benth. et Hook.f. (白芷)	1	40	2					Y					Y	Y	
E	*Vitex trifolia* L. var. *simplicifolia* Cham. (蔓荊子)	1	34	2	Y									Y		Y
E	*Aconitum carmichaelii* Debx. (附子)	7	29	2		Y		Y		Y						
E	*Zanthoxylum schinifolium* Sieb. et Zucc. (蜀椒)	7	26	2				Y	Y	Y				Y		
E	*Carthamus tinctorius* L. (紅花)	12	25	1	Y	Y										
E	*Salvia miltiorrhiza* Bge. (丹蔘)	12	25	1	Y	Y	Y									
E	*Saposhnikovia divaricata* (Turcz.) Schischk. (防風)	1	23	2	Y			Y								Y

*BL* Bladder meridian, *GB* Gall bladder meridian, *HT* Heart meridian, *KI* Kidney meridian, *LI* Large intestine meridian, *LR* Liver meridian, *LU* Lung meridian, *PC* Pericardium meridian, *SI* Small intestine meridian, *SP* Spleen meridian, *ST* Stomach meridian, *TE* Triple energizer meridian

*Angelica sinensis* (Oliv.) Dlels (當歸) was the 2nd most commonly used medicinal herb in the internal medicines category and the 6th most commonly used in the external application category

*Ligusticum chuanxiong* Hort. (川芎) was the 4th most commonly used medicinal herb in the internal medicines category and the 4th most commonly used in the external application category

*E*, External application, *I* Internal Medicine, *I&E* Commonly used in external application and internal medicine

Category of each medicinal herb: 1, Exterior-releasing medicinal (解表藥); 2, Heat-clearing medicinal (淸熱藥); 4, Wind-dampness dispelling medicinal (祛風濕藥); 5, Dampness-resolving medicinal (化濕藥); 6, Dampness-draining diuretic medicinal (利水滲濕藥); 7, Interior-warming medicinal (溫裏藥); 8, *Qi*-regulating medicinal (理氣藥); 9, Digestant medicinal (消食藥); 11, Hemostatic medicinal (止血藥); 12, Blood-activating and stasis-dispelling medicinal (活血祛瘀藥); 13, Cough-suppressing and panting-calming medicinal (止咳平喘藥); 14, Tranquillizing medicinal (安神藥); 15, Liver-pacifying medicinal (平肝藥); 17, Tonifying and replenishing medicinal (補益藥); 18, Astringent medicinal (收澁藥)

### Association rule mining results for two-herb and three-herb combinations

The frequency trend of the 258 herbs in the 312 internal alopecia prescriptions were analyzed using the a priori ARM method to elucidate whether certain herbs are used more frequently in combinations of two or three. The top 10 association rules between two herbs are described in Table 2. The association of *Polygonum multiflorum* Thunb. (何首烏) and *Angelica sinensis* (Oliv.) Dlels (當歸) had the highest support, with 38.5%. The six possible pairs from a group of 4 herbs—*Polygonum multiflorum* Thunb. (何首烏), *Angelica sinensis* (Oliv.) Dlels (當歸), *Ligusticum chuanxiong* Hort. (川芎), and *Rehmannia glutinosa* Libosch. (Prepared) (熟地黃)—were listed as the top 6 association rules, indicating that these four herbs are the most commonly prescribed herbs, and that they are frequently used together. Other rules in the top 10 list contained *Ligustrum lucidum* Ait. (女貞子), *Lycium barbarum* L. (枸杞子), and *Eclipta prostrata* L. (旱蓮草) as additional combination herbs.

**Table 2:** Association rule mining results (Length of herb set = 2)

Associated Herbs	support	confidence	lift	frequency
*Polygonum multiflorum* Thunb. (何首烏), *Angelica sinensis* (Oliv.) Dlels (當歸)	0.385	0.702	1.251	120
*Ligusticum chuanxiong* Hort. (川芎), *Angelica sinensis* (Oliv.) Dlels (當歸)	0.343	0.811	1.479	107
*Rehmannia glutinosa* Libosch. (Prepared) (熟地黃), *Angelica sinensis* (Oliv.) Dlels (當歸)	0.304	0.693	1.265	95
*Rehmannia glutinosa* Libosch. (Prepared) (熟地黃), *Polygonum multiflorum* Thunb. (何首烏)	0.279	0.635	1.132	87
*Ligusticum chuanxiong* Hort. (川芎), *Polygonum multiflorum* Thunb. (何首烏)	0.263	0.621	1.108	82
*Ligusticum chuanxiong* Hort. (川芎), *Rehmannia glutinosa* Libosch. (Prepared) (熟地黃)	0.253	0.598	1.363	79
*Ligustrum lucidum* Ait. (女貞子), *Polygonum multiflorum* Thunb. (何首烏)	0.231	0.750	1.337	72
*Lycium barbarum* L. (枸杞子), *Polygonum multiflorum* Thunb. (何首烏)	0.221	0.784	1.398	69
*Ligustrum lucidum* Ait. (女貞子), *Eclipta prostrata* L. (旱蓮草)	0.221	0.719	2.520	69
*Eclipta prostrata* L. (旱蓮草), *Polygonum multiflorum* Thunb. (何首烏)	0.215	0.753	1.342	67

The top 10 association rules for three-herb combinations are described in Table 3. The result again showed the importance of four main herbs. Of the four possible combinations of three herbs from among the four main herbs—*Polygonum multiflorum* Thunb. (何首烏), *Angelica sinensis* (Oliv.) Dlels (當歸), *Ligusticum chuanxiong* Hort. (川芎), and *Rehmannia glutinosa* Libosch. (Prepared) (熟地黃)—three combinations were listed in the top 4 association rules, with support of 23.4, 21.5, and 21.2%, respectively. One other three-herb combination of the four main herbs, excluding *Angelica sinensis* (Oliv.) Dlels (當歸), was listed as the No. 8 association rule.

**Table 3:** Association rule mining results (Length of herb set = 3)

Associated Herbs	support	confidence	lift	frequency
*Ligusticum chuanxiong* Hort. (川芎), *Polygonum multiflorum* Thunb. (何首烏), *Angelica sinensis* (Oliv.) Dlels (當歸)	0.234	0.890	1.624	73
*Rehmannia glutinosa* Libosch. (Prepared) (熟地黃), *Polygonum multiflorum* Thunb. (何首烏), *Angelica sinensis* (Oliv.) Dlels (當歸)	0.215	0.770	1.405	67
*Rehmannia glutinosa* Libosch. (Prepared) (熟地黃), *Ligusticum chuanxiong* Hort. (川芎), *Angelica sinensis* (Oliv.) Dlels (當歸)	0.212	0.835	1.524	66
*Eclipta prostrata* L. (旱蓮草), *Polygonum multiflorum* Thunb. (何首烏), *Ligustrum lucidum* Ait. (女貞子)	0.167	0.776	2.522	52
*Paeonia lactiflora Pall.* (白芍藥), *Rehmannia glutinosa* Libosch. (Prepared) (熟地黃), *Angelica sinensis* (Oliv.) Dlels (當歸)	0.154	0.842	1.536	48
*Ligustrum lucidum* Ait. (女貞子), *Polygonum multiflorum* Thunb. (何首烏), *Angelica sinensis* (Oliv.) Dlels (當歸)	0.154	0.667	1.216	48
*Lycium barbarum* L. (枸杞子), *Polygonum multiflorum* Thunb. (何首烏), *Angelica sinensis* (Oliv.) Dlels (當歸)	0.151	0.904	1.611	47
*Ligusticum chuanxiong* Hort. (川芎), *Polygonum multiflorum* Thunb. (何首烏), *Rehmannia glutinosa* Libosch. (Prepared) (熟地黃)	0.151	0.573	1.305	47
*Paeonia lactiflora* Pall. (白芍藥), *Ligusticum chuanxiong* Hort. (川芎), *Angelica sinensis* (Oliv.) Dlels (當歸)	0.147	0.885	1.614	46
*Angelica sinensis* (Oliv.) Dlels (當歸), *Cuscuta chinensis* Lam. (菟絲子), *Polygonum multiflorum* Thunb. (何首烏)	0.144	0.804	1.433	45

*Paeonia lactiflora* Pall. (白芍藥) did not appear in the top 10 association rules of two-herb combinations, but it was listed as a member of three-herb combinations in association rule Nos. 5 and 9. *Cuscuta chinensis* Lam. (菟絲子) was also not shown in the top 10 association rules of two-herb combinations, but it did appear among the top 10 association rules of three-herbs combinations.

### Modularity analysis of herb networks and characteristic of modules

**Fig. 2:**
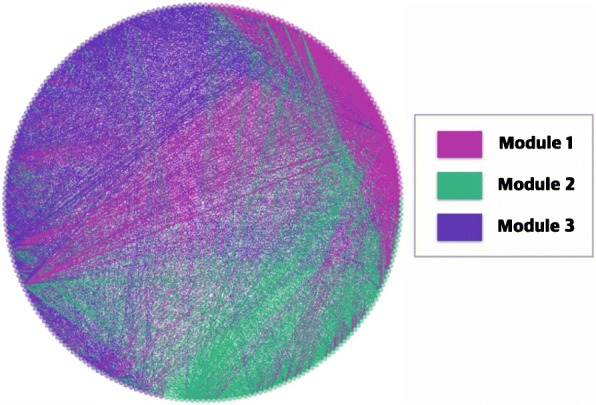
Herb network of alopecia prescription based on modularity analysis

Based on the frequency of co-occurrences of herb pairs, we constructed a weighted unidirectional network, assigning “frequency of co-occurrence” as a weight value of the connection. We then performed a modularity analysis on the weighted graph using the Louvain method. The results showed that the network could be divided into three modules, with a modularity value of 0.141. The number of herbs comprising each module were 58, 86, and 111, respectively (Fig. 2). Unconnected single herbs were excluded from the modularity analysis. The top 20 most frequent herbs in each module are described in Table 4.

**Table 4:** Meridian tropism and category of the top 20 herbs in each network module

M	Medicinal Herb	W	Category	LR (肝)	HT (心)	PC (心包)	SP (脾)	LU (肺)	KI (腎)	GB (膽)	SI (小腸)	TE (三焦)	ST (胃)	LI (大腸)	BL (膀胱)
1	*Polygonum multiflorum* Thunb. (何首烏)	1175	17	Y					Y						
1	*Angelica sinensis* (Oliv.) Dlels (當歸)	1100	17	Y	Y		Y								
1	*Rehmannia glutinosa* Libosch. (Prepared) (熟地黃)	956	17	Y					Y						
1	*Ligusticum chuanxiong* Hort. (川芎)	904	12	Y		Y				Y					
1	*Ligustrum lucidum* Ait. (女貞子)	720	17	Y					Y						
1	*Eclipta prostrata* L. (旱蓮草)	644	17	Y					Y						
1	*Lycium barbarum* L. (枸杞子)	622	17	Y				Y	Y						
1	*Rehmannia glutinosa* Libosch. (生地黄)	603	2	Y	Y				Y						
1	*Cuscuta chinensis* Lam. (菟絲子)	591	17	Y			Y		Y						
1	*Astragalus membranaceus* (Fisch.) Bge. (黃芪)	564	17				Y	Y							
1	*Paeonia lactiflora* Pall. (白芍藥)	535	17	Y			Y								
1	*Salvia miltiorrhiza* Bge. (丹蔘)	484	12	Y	Y	Y									
1	*Morus alba* L. (桑椹)	481	17	Y	Y				Y						
1	*Sesamum indicum* L. (黑芝麻)	441	17	Y					Y					Y	
1	*Platycladus orientalis* (L.) Franco (側柏葉)	355	11	Y				Y						Y	
1	*Gastrodia elata* Bl. (天麻)	288	15	Y											
1	*Carthamus tinctorius* L. (紅花)	284	12	Y	Y										
1	*Codonopsis pilosula* (Franch.) Nannf. (唐蔘)	232	17				Y	Y							
1	*Chaenomeles speciosa* (Sweet) Nakai (木瓜)	230	4	Y			Y								
1	*Polygonatum sibiricum* Red. (黃精)	215	17				Y	Y	Y						
1	Total	–	**–**	17	5	2	7	5	10	1	0	0	0	2	0
2	*Saposhnikovia divaricata* (Turcz.) Schischk. (防風)	85	1	Y			Y								Y
2	*Paeonia suffruticosa* Andr. (牧丹皮)	83	2	Y	Y				Y						
2	*Panax ginseng* C.A.Mey. (人蔘)	82	8				Y	Y	Y						
2	*Platycladus orientalis* (L.) Franco (柏子仁)	62	14		Y				Y					Y	
2	*Zanthoxylum schinifolium* Sieb. et Zucc. (蜀椒)	61	7				Y	Y	Y				Y		
2	*Dendrobium nobile* Lindl. (石斛)	60	17						Y				Y		
2	*Cinnamomum cassia* Presl (肉桂)	60	7	Y	Y		Y		Y						
2	*Angelica dahurica* (Fisch. ex Hoffm.) Benth. et Hook.f. (白芷)	57	1					Y					Y	Y	
2	*Achyranthes bidentata* Bl. (牛膝)	56	12	Y					Y						
2	*Eucommia ulmoides* Oliv. (杜沖)	51	17	Y					Y						
2	*Sophora flavescens* Ait. (苦蔘)	48	2	Y	Y								Y	Y	Y
2	*Schisandra chinensis* (Turcz.) Baill. (五味子)	46	18		Y			Y	Y						
2	*Ligusticum sinense* Oliv. (藁本)	44	1												Y
2	*Asarum heterotropoides* Fr.Schmidt var. *mandshuricum* (Maxim.) Kitag. (細辛)	42	1					Y	Y						
2	*Cistanche deserticola* Y.C.Ma (肉蓯蓉)	40	17						Y					Y	
2	*Zingiber officinale* Rosc. (乾薑)	39	7		Y		Y	Y					Y		
2	*Tribulus terrestris* L. (蒺藜)	37	15												
2	*Schizonepeta tenuifolia* Briq. (荊芥)	37	1	Y				Y							
2	*Lycopus lucidus* Turcz. var. *hirtus* Regel (澤蘭)	37	12	Y			Y								Y
2	*Selaginella tamariscina* (Beauv.) Spring (卷柏)	36	12	Y						Y					
2	Total	–	**–**	9	6	0	6	7	11	1	0	0	5	4	4
3	*Poria cocos* (Schw.) Wolf (茯苓)	469	6		Y		Y		Y						
3	*Glycyrrhiza uralensis* Fisch. (甘草)	353	8												
3	*Atractylodes macrocephala* Koidz. (白朮)	294	8				Y						Y		
3	*Alisma orientale* (Sam.) Juzep. (澤瀉)	205	6						Y						Y
3	*Bupleurum chinense* DC. (柴胡)	165	1	Y			Y			Y					
3	*Crataegus pinnatifida* Bge. (山楂)	163	9	Y			Y						Y		
3	*Dioscorea opposita* Thunb. (山藥)	155	8				Y	Y	Y						
3	*Cornus officinalis* Sieb. et Zucc. (山茱萸)	140	18	Y					Y						
3	*Citrus reticulata* Blanco (陳皮)	129	8				Y	Y					Y		
3	*Scutellaria baicalensis* Georgi (黃芩)	129	2	Y				Y		Y	Y		Y	Y	
3	*Coix lacryma-jobi* L. var. *ma-yuen* (Roman.) Stapf (薏苡仁)	125	6				Y	Y					Y		
3	*Polygonum multiflorum* Thunb. (夜交藤)	119	14	Y	Y										
3	*Dictamnus dasycarpus* Turcz. (白鮮皮)	114	2				Y						Y		Y
3	*Pinellia ternata* (Thunb.) Breit. (半夏)	99	13				Y	Y					Y		
3	*Zingiber officinale* Rosc. (生薑)	98	1				Y	Y					Y		
3	*Gardenia jasminoides* Ellis (梔子)	95	2	Y	Y			Y				Y	Y		
3	*Artemisia capillaris* Thunb. (茵蔯蒿)	89	6	Y			Y			Y			Y		
3	*Ziziphus jujuba* Mill. (大棗)	89	8				Y						Y		
3	*Plantago asiatica* L. (車前子)	82	6	Y				Y	Y						
3	*Atractylodes lancea* (Thunb.) DC. (蒼朮)	80	5				Y						Y		
3	Total	–	**–**	8	3	0	13	8	5	3	1	1	12	1	2

Category of each medicinal herb: 1, Exterior-releasing medicinal (解表藥); 2, Heat-clearing medicinal (淸熱藥); 4, Wind-dampness dispelling medicinal (祛風濕藥); 5, Dampness-resolving medicinal (化濕藥); 6, Dampness-draining diuretic medicinal (利水滲濕藥); 7, Interior-warming medicinal (溫裏藥); 8, *Qi*-regulating medicinal (理氣藥); 9, Digestant medicinal (消食藥); 11, Hemostatic medicinal (止血藥); 12, Blood-activating and stasis-dispelling medicinal (活血祛瘀藥); 13, Cough-suppressing and panting-calming medicinal (止咳平喘藥); 14, Tranquillizing medicinal (安神藥); 15, Liver-pacifying medicinal (平肝藥); 17, Tonifying and replenishing medicinal (補益藥); 18, Astringent medicinal (收澁藥)

*M* Module, *W* Weighted degree

Meridian tropism: *BL* Bladder meridian, *GB* Gall bladder meridian, *HT* Heart meridian, *KI* Kidney meridian, *LI* Large intestine meridian, *LR* Liver meridian, *LU* Lung meridian, *PC* Pericardium meridian, *SI* Small intestine meridian, *SP* Spleen meridian, *ST*, Stomach meridian, *TE* Triple energizer meridian

**Fig. 3:**
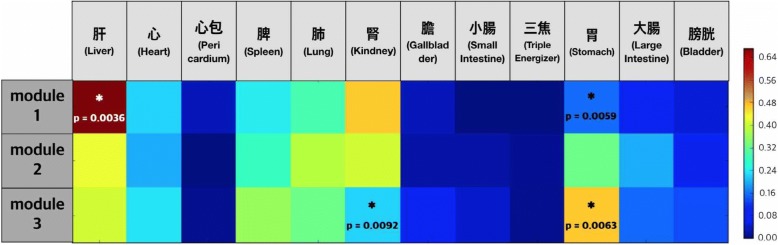
Occurrence ratio of associated meridians of herbs within each module

The frequency trend for meridian tropism in each module is described in Fig. 3. Significantly high and low meridian tropism frequency ratios were found in each module using the permutation test. Module 1 had significantly more herbs that preferred “Liver,” while Module 3 had significantly more herbs that preferred “Stomach.”

## Discussion

We systematically searched literature for alopecia treatment formulas. The following herbs were frequently used in internal medicine: *Polygonum multiflorum* Thunb., *Angelica sinensis* (Oliv.) Dlels, *Rehmannia glutinosa* Libosch. (Prepared), and *Ligusticum chuanxiong* Hort. Conversely, *Platycladus orientalis* (L.) Franco, *Angelica dahurica* (Fisch. ex Hoffm.) Benth. et Hook.f., *Vitex trifolia* L. var. *simplicifolia* Cham., and *Ligusticum chuanxiong* Hort. were frequently used in external applications. Internal medicine and external application differed in terms of pharmacological efficacy and meridian tropism. Using the ARM method, the most frequently used two-herb combinations were (1) *Polygonum multiflorum* Thunb. and *Angelica sinensis* (Oliv.) Dlels, and (2) *Ligusticum chuanxiong* Hort. and *Rehmannia glutinosa* Libosch. (Prepared). *Ligusticum chuanxiong* Hort., *Polygonum multiflorum* Thunb., and *Angelica sinensis* (Oliv.) Dlels was the most frequently used three-herb combination. Using the network analysis method, we classified the herbs into three modules. The meridian entry (歸經) of many herbs in module 1 was “Liver,” whereas that in module 3 was “Stomach”.

The meridian tropism theory is important in traditional East Asian medicine—both pharmacologically and in clinical practice. [74] According to meridian tropism theory, medicinal herbs have a certain *qi* and flavor (氣味), and they exhibit curative effects on selected meridians. Therefore, depending on whether it belongs to the viscera or bowel group (臟腑), each medicinal herb is mainly used in a specific region. In other words, meridian tropism is a theory of the orientation of drug action. [75] Several experimental studies have presented evidence for meridian tropism theory. [74–76]

In the present study, the internal medicine group—*Polygonum multiflorum* Thunb., *Angelica sinensis* (Oliv.) Dlels, *Rehmannia glutinosa* Libosch. (Prepared), *Ligusticum chuanxiong* Hort., and *Poria cocos* (Schw.) Wolf—appeared in order. When we analyzed the top 10 medicinal herbs in the internal medicine group, every herb except for *Ligusticum chuanxiong* Hort. belonged to the viscera meridian (臟), not the bowel meridian (腑). [12] In the external application group—*Platycladus orientalis* (L.) Franco, *Angelica dahurica* (Fisch. ex Hoffm.) Benth. et Hook.f., *Vitex trifolia* L. var. *simplicifolia* Cham., *Ligusticum chuanxiong* Hort., and *Aconitum carmichaelii* Debx.—appeared in order. Six herbs in external application group belong to the bowel meridian. [12] These differences in meridian tropism between internal and external medicine may be associated with the drug absorption pathways or medicinal guide herb (引經藥). [77] The category of each medicinal herb also differed. In the internal medicine group, six herbs belonged to the tonifying and replenishing medicinal category. However, in the external application group, three herbs were exterior-releasing medicinals, and three were blood-activating and stasis-dispelling medicinals (Table 1).

We identified frequently used two-herb and three-herb set combinations (Tables 2 & 3). *Polygonum multiflorum* Thunb., *Angelica sinensis* (Oliv.) Dlels, *Ligusticum chuanxiong* Hort. and *Rehmannia glutinosa* Libosch. (Prepared) are the main herbs used in alopecia treatment. The six two-herb combinations of the four main herbs were the top six combinations of two-herb sets. These four main herbs were also important in the three-herb sets. However, the three-herb combination of *Polygonum multiflorum* Thunb., *Ligusticum chuanxiong* Hort., and *Rehmannia glutinosa* Libosch. (Prepared) occupied the relatively low 8th place. *Paeonia lactiflora* Pall. did not appear in the two-herb sets, but it was frequently observed in the three-herbs sets, indicating that this herb is used as an adjunct in alopecia treatment.

Interestingly, the lift value of the *Ligustrum lucidum* Ait. and *Eclipta prostrata* L. combination was higher than the frequency and support values, and these herbs often appeared together with *Polygonum multiflorum* Thunb. or *Angelica sinensis* (Oliv.) Dlels. That said, all the medicinal herbs appeared frequently. In contrast, in the case of *Ligustrum lucidum* Ait. and *Eclipta prostrata* L., the lift value was higher than the frequency of each medicinal herb, indicating that *Ligustrum lucidum* Ait. and *Eclipta prostrata* L. are usually prescribed together. The herbal formula name of the *Ligustrum lucidum* Ait. and *Eclipta prostrata* L. combination is Yijihwan (二至丸). It has antioxidant activity and has been prescribed for hair loss in clinical practice. [78] Among the three-herb sets, the lift value of the *Ligustrum lucidum* Ait., *Eclipta prostrata* L., and *Polygonum multiflorum* Thunb. combination was also relatively high.

When we use the ARM method, the number of herbs that comprise each herb set should be determined in advance. For this reason we only identified frequently used two-herb and three-herb sets (Tables 2 & 3), and we used network analysis to assess the relationships of all medicinal herbs used to treat hair loss, regardless of the number of herbs in the set (Figs. 2 & 3). We reviewed previous literature regarding pattern identification in alopecia. [6, 7, 79–81] Blood heat engendering wind (血熱生風), blood stasis due to *qi* stagnation (氣滯血瘀), dual deficiency of *qi* and blood (氣血兩虛), liver-kidney depletion (肝腎不足), and spleen-stomach dampness-heat (脾胃濕熱) were the major pattern identifications in alopecia. Pathology was classified in terms of the viscera and bowels (臟腑) theory or the *qi* and blood (氣血) theory.

According to our network analysis, Module 1 herbs affect the “Liver” meridian more and seem to tonify *qi* and blood. [82] Module 3 herbs belong to the “Stomach” meridian more and seem to help digestion and absorption. Module 2 herbs seem to act on body surfaces, and they tend to be used externally, although further research is needed in this regard. These modules were similar to the traditional pattern identification framework derived from alopecia literature reviews. [6, 7, 79–81] In the present study, we reconfirmed the that Module 1 comprises tonifying “Liver” and “Kidney” strategies, and that Module 3 belongs more to the “Stomach” meridian, indicating that treatment of digestion and absorption are important in alopecia treatment.

Among the top 10 herbs in the internal medication group, none belonged to Module 2. All herbs except for *Poria cocos* (Schw.) Wolf and *Glycyrrhiza uralensis* Fisch. belonged to Module 1 and the “Liver” meridian (Table 1). All herbs except for *Ligusticum chuanxiong* Hort. belonged to the viscera (臟) group and not the bowel (腑) group. In contrast, of the top 10 herbs in the external application group, five belonged to module 2. They also affected bowel meridians such as the “Stomach,” “Large intestine,” and “Urinary bladder.” Thus, it may be that Module 2 is associated with external application, but further study will be needed, as we only conducted network analysis on the internal medicine group. Many of herbs in Module 3 belong more to the “Stomach” meridian. However, there were not module 3 medicinal herb in the internal and external groups top 10 herbs, with the exception of *Poria cocos* (Schw.) Wolf and *Glycyrrhiza uralensis* Fisch., indicating that treatment of digestion and absorption, which are related to Module 3, may be an adjunctive strategy in traditional Asian medicine. However, further research is needed in this regard.

Additional analysis was conducted on the top 20 medicinal herbs of each module (Table 4). In Module 1, 13 herbs were tonifying and replenishing medicinals (補益藥), mostly oriented towards the “Liver” and “Kidney” meridians. Therefore, Module 1 herbs are characterized as tonifying the “Liver” and “Kidney” meridians. Five dampness-draining diuretic medicinals (利水滲濕藥) and five *Qi*-regulating medicinals (理氣藥) occupy half of Module 3. Most of these were oriented towards the “Spleen” and “Stomach” meridians. Therefore, Module 3 herbs are related to digestive function.

Module 2 comprised five exterior-releasing medicinals (解表藥), three interior-warming medicinals (溫裏藥), and three blood-activating and stasis-dispelling medicinals (活血祛瘀藥). [12, 13] Thus, Module 2 was apparently associated with excretion and divergence., Presumably, Module 2 herbs act on the body surface or are external medicines, although further research is needed in this regard.

Existing studies on pattern identification have taken a top-down theoretical approach. In contrast, the present research adopted a practical, bottom-up approach based on formulas that are prescribed in clinical practice. We conducted this novel approach to pattern identification by carrying out a network analysis of medicinal herbs used in alopecia treatment. We rediscovered the classical pattern identification of alopecia treatment, and we suggest that clinicians adopt a “Liver” or “Stomach”-oriented approach to alopecia treatment.

The current research had several strengths. To our knowledge, this was the first study that used bioinformatics methods and searched Chinese, English, and Korean databases to assess which medicinal herbs have been used to treat alopecia. We adopted a practical network analysis approach based on formulas that are frequently used in clinical practice, rather than a theoretical/literature approach. Using this method, we explored the frequency, combination patterns, and meridian tropism of medicinal herbs used in alopecia treatment. We also classified herbs into three modules, confirming the value of classical pattern identification and the meridian tropism theory. Moreover, we explored the pathology of alopecia from the perspective of traditional east Asian medicine.

Our data mining methodology, which employed ARM and network analysis, also had several strengths. Firstly, in the ARM method, the number of herbs comprising the combination must be determined in advance. To overcome such shortcomings, we used network analysis to look at the overall combination pattern of medicinal herbs without limiting the number of herbs in the combination. Secondly, previous top-down research based on ancient literature has offered hypotheses about the pattern identification category of alopecia. In contrast, our bottom-up study categorized herbs into three modules based on the combination patterns of the formula. Lastly, previous research was limited in that it could only “qualitatively” interpret the characteristics of medicinal herbs or formulas used in alopecia treatment. We overcame this limitation by extracting significant “quantitative” characteristics using the permutation test.

Our research also had several limitations. The present study was based on the frequency of formulas used in clinical practice and literature. For this reason, we could not evaluate new candidate medicinal herbs emerging from recent clinical/experimental studies, neither could we reflect the importance of medicinal herb dose in each formula. Relatedly, we did not evaluate the clinical effectiveness of each formula in our study. Further clinical/experimental studies are needed to assess whether the classifications derived from our research have real meaning. Meridian tropism theory is controversial and may not accurately reflect the characteristics of each medicinal herb. Finally, we did not analyze external medicine, and the heterogeneity within Module 2 was not completely resolved.

The present research raises several indications for future research. We should analyze externally applied alopecia treatments, and we need to conduct a proof-of-concept study to corroborate our research. Using network pharmacologic analysis of medicinal herbs in each module, a hair loss mechanism could be identified based on meridian tropism theory (traditional medicine theory). Such studies may also indicate the pharmacological mechanism of hair loss treatment (western medicine theory). Multi-component, multi-target concepts are essential in herbal medicine pharmacology. Thus, we could propose new research methodology based on the techniques used in the present study. This methodology could be utilized to develop new hair loss drugs from natural products.

## Conclusions

We identified the frequency and characteristics of medicinal herbs used in alopecia treatment. The most frequently used two-herb combination in alopecia treatment consisted of *Polygonum multiflorum* Thunb. and *Angelica sinensis* (Oliv.) Dlels. The most frequently used three-herb combination was *Polygonum multiflorum* Thunb., *Angelica sinensis* (Oliv.) Dlels, and *Ligusticum chuanxiong* Hort. Based on the meridian tropism theory, we used network analysis to identify three modules of herbs that can treat alopecia. We found a “Liver”-oriented module and a “Stomach”-oriented module, and confirmed the value of classical meridian tropism theory and pattern identification. However, further clinical/experimental study is needed to prove the significance of this concept and methodology.

## Additional file

Additional file 1: Search strategy according to database. Individual search strategies for each database (DOCX 15 kb)

## Abbreviations

ARM: Association Rule Mining
CAM: Complementary and Alternative Medicine
CENTRAL: Central Register of Controlled Trials
CNKI: China National Knowledge Infrastructure Database
FDA: USA., the Food and Drug Administration of the United States
KIOM: Korea Institute of Oriental Medicine
OASIS: Oriental Medicine Advanced Searching Integrated System
TCM: Traditional Chinese Medicine
