# Supplementary material for: Exploring the combination and modular characteristics of herbs for alopecia treatment in traditional Chinese medicine: an association rule mining and network analysis study
Source: BMC Complement Altern Med. 2018 Jul 4;18:204. doi: 10.1186/s12906-018-2269-7 (PMC6030800; doi:10.1186/s12906-018-2269-7)
Supplement: Supplementary file 1 — Search strategy according to database. Individual search strategies for each database (DOCX 15 kb) [file 12906_2018_2269_MOESM1_ESM.docx]

# Medline (via Pubmed)

#1 "Alopecia"[Mesh] 11945

#2 "Alopecia Areata"[Mesh] 2768

#3 "Diffuse alopecia" [Supplementary Concept] 41

#4 “androgenic alopecia” 303

#5 "androgenetic alopecia” 18994

#6 “alopecia androgenetica” 18

#7 ((hair loss OR baldness OR alopecia) AND (androgen OR androgens)) 1056

#8 “female pattern hair loss”) 176

#9 “female baldness” 4

#10 (“female pattern” AND hair loss) 220

#11 alopecia totalis 315

#12 Alopecia Areata 3635

#13 alopecia universalis 335

#14 alopecia celsi 230

#15 ophiasis 47

#16 nonscarring hair loss 172

#17 Alopecia 18994

#18 hair loss 27713

#19 (loss*) AND hair) 11696

#20 Telogen effluvium 253

#21 Anagen effluvium 86

#22 or/#1 - #21 28029

#23 "Plants, Medicinal"[Mesh]) 55523

#24 "Herbal Medicine"[Mesh] 1697

#25 "Medicine, Chinese Traditional"[Mesh]) 15170

#26 herbal medicine 32650

#27 herbal decoction 2947

#28 herbal granule 654

#29 or/#23-#28 94318

#30 #22 AND #29 81

# Cochrane (CENTRAL Library)

#1 MeSH descriptor: [Alopecia] explode all trees 382

#2 MeSH descriptor: [Alopecia Areata] explode all trees 85

#3 Diffuse alopecia:ti,ab,kw (Word variations have been searched) 36

#4 androgenic alopecia:ti,ab,kw (Word variations have been searched) 49

#5 androgenetic alopecia:ti,ab,kw (Word variations have been searched) 175

#6 hair loss:ti,ab,kw (Word variations have been searched) 644

#7 baldness:ti,ab,kw (Word variations have been searched) 70

#8 alopecia:ti,ab,kw (Word variations have been searched) 2458

#9 female pattern hair loss:ti,ab,kw (Word variations have been searched) 35

#10 female baldness:ti,ab,kw (Word variations have been searched) 11

#11 alopecia totalis:ti,ab,kw (Word variations have been searched) 30

#12 Alopecia Areata:ti,ab,kw (Word variations have been searched) 210

#13 alopecia universalis:ti,ab,kw (Word variations have been searched) 21

#14 alopecia celsi:ti,ab,kw (Word variations have been searched) 0

#15 ophiasis:ti,ab,kw (Word variations have been searched) 1

#16 nonscarring hair loss:ti,ab,kw (Word variations have been searched) 2

#17 Telogen effluvium:ti,ab,kw (Word variations have been searched) 8

#18 Anagen effluvium:ti,ab,kw (Word variations have been searched) 5

#19 {or #1-#18} 2906

#20 MeSH descriptor: [Plants, Medicinal] explode all trees 969

#21 MeSH descriptor: [Herbal Medicine] explode all trees 67

#22 MeSH descriptor: [Medicine, Chinese Traditional] explode all trees 1095

#23 herbal medicine:ti,ab,kw (Word variations have been searched) 2980

#24 herbal decoction:ti,ab,kw (Word variations have been searched) 489

#25 herbal granule:ti,ab,kw (Word variations have been searched) 339

#26 {or #20-#25} 4780

#27 #19 and #26 14

# EMBASE

#1 alopecia'/exp OR alopecia 46022

#2 'alopecia'/exp OR alopecia AND areata 5534

#3 'diffuse alopecia'/exp OR 'diffuse alopecia' 42759

#4 'androgenic alopecia'/exp OR 'androgenic alopecia' 470

#5 'androgenetic alopecia'/exp OR 'androgenetic alopecia' 2628

#6 'hair loss'/exp OR 'hair loss' 12469

#7 'baldness'/exp OR baldness 42926

#8 'female pattern hair loss'/exp OR 'female pattern hair loss' 256

#9 'female baldness' 8

#10 'alopecia totalis'/exp OR 'alopecia totalis' 426

#11 'alopecia universalis'/exp OR 'alopecia universalis' 442

#12 'alopecia celsi' 0

#13 ophiasis 72

#14 'nonscarring hair loss' 53

#15 'telogen effluvium'/exp OR 'telogen effluvium' 427

#16 'telogen'/exp OR telogen AND effluvium 446

#17 or/#1-#16 54269

#18 'plants, medicinal'/exp OR 'plants, medicinal' 189096

#19 'medicinal plants'/exp OR 'medicinal plants' 24452

#20 'herbal medicine'/exp OR 'herbal medicine' 26778

#21 'medicine, chinese traditional'/exp OR 'medicine, chinese traditional' 30442

#22 'herbal medicine'/exp OR 'herbal medicine' 26778

#23 'herbal decoction' 216

#24 'herbal granule' 8

#25 or/ #18 - #24 233630

#26 #17 and #25 561

#27 #26 AND [humans]/lim AND [clinical study]/lim AND [embase]/lim 191

# CNKI

#1 (脂溢性)脱发 OR 全秃OR 普秃 OR 斑秃 OR 雄激素源性脱发OR 休止期脱发

Search in Results

#2 中草药 OR 中药 OR 中成藥 OR 中药汤剂 OR 中药颗粒 905

#3 After 2016 72
